# Geroscience Brings New Focus on the Life-Course Approach to Health in Humans

**DOI:** 10.1093/geroni/igaa057.2914

**Published:** 2020-12-16

**Authors:** Luigi Ferrucci

**Affiliations:** National Institute on Aging, Bethesda, Maryland, United States

## Abstract

The geroscience paradigm stands on two assumptions: 1. Major changes in health are caused by accelerated aging; 2. The rate of aging can be modified by interventions with significant effects on health span. These simple statements change dramatically the portrayal of aging research in medicine. In the past, aging was considered fixed and irreversible, and the study of aging a speculative science, closer to anthropology than to physiology. However, if biological aging is the root cause of chronic disease and if the rate of aging can be changed, then the study of aging becomes the most important branch of medical research. The last twenty years of research support this view and suggest that the rate of aging is set during early development but is modifiable by appropriate interventions. These findings suggest that the architecture of disease development and subsequent loss of function may be more easily influenced by early interventions.

